# The Impact of Training Intervention on Levels of Indicator Bacteria and Prevalence of Selected Pathogens in Raw Milk From Smallholder Women Dairy Farmers in Central Ethiopia

**DOI:** 10.1016/j.jfp.2024.100446

**Published:** 2025-02-03

**Authors:** Achenef Melaku Beyene, Seleshe Nigatu, Juan C. Archila-Godinez, Kebede Amenu, Barbara Kowalcyk, Desalegne Degefaw, Binyam Mogess, Baye Gelaw, Mucheye Gizachew, Araya Mengistu, Ahmed G. Abdelhamid, James Barkley, Ahmed Yousef

**Affiliations:** 1 Department of Medical Microbiology, College of Medicine and Health Sciences, University of Gondar, Gondar, Ethiopia; 2 Department of Veterinary Epidemiology and Public Health, College of Veterinary Medicine and Animal Sciences, University of Gondar, Gondar, Ethiopia; 3 Center for Foodborne Illness Research and Prevention, Department of Food Science and Technology, College of Food Agriculture and Environmental Sciences, The Ohio State University, Columbus, Ohio, USA; 4 Department of Food Science and Technology, College of Food Agriculture and Environmental Sciences, The Ohio State University, Columbus, Ohio, USA; 5 Department of Environmental and Occupational Health, Milken Institute School of Public Health, George Washington University, Washington, District of Columbia, USA; 6 Animal and Human Health Program, International Livestock Research Institute, Addis Ababa, Ethiopia; 7 College of Veterinary Medicine and Agriculture, Addis Ababa University, Bishoftu, Ethiopia; 8 Translational Data Analytics Institute, The Ohio State University, Columbus, OH, USA; 9 Department of Exercise and Nutrition Sciences, Milken Institute School of Public Health, George Washington University, Washington, District of Columbia, USA; 10 The Ohio State University, Global One Health Initiative, Addis Ababa, Ethiopia; 11 Department of Food Science and Human Nutrition, Michigan State University, East Lansing, Michigan, USA

**Keywords:** Central Ethiopia, Indicator bacteria, Pathogenic bacteria, Raw milk, Training, Women dairy farmers

## Abstract

Contamination of milk is a serious public health risk, particularly in developing countries such as Ethiopia. Training is a tool for improving the quality and safety of milk. However, its effect on the microbial quality and safety of milk has not been well documented. This study assessed the impact of training interventions on the microbial quality and safety of milk, with a specific focus on smallholder dairy farms. The study was conducted from January to June 2022 in four locations in Central Ethiopia. Milk samples were collected from 120 dairy farmers one week before and four to six weeks after the training and analyzed for total coliforms, thermotolerant coliforms, *Escherichia coli*, Shiga toxin-producing *E. coli* (STEC), *Salmonella enterica,* and *Campylobacter jejuni*. Total and thermotolerant coliforms were determined by the Most Probable Number (MPN) method; categorized as high (≥1,001 MPN/ml), medium (101 10^3^ MPN/ml), low (21–10^2^ MPN/ml), and very low (≤20 MPN/ml); and analyzed using generalized linear mixed models. Prevalence of *E. coli*, STEC, *S. enterica*, and *C. jejuni* in raw milk samples were 67, 12, 3, and 4% pretraining and 45, 4, 3, and 2% posttraining, respectively. The prevalence of *E. coli* (*p* = 0.0389) and STEC (*p* = 0.0005) was significantly lower posttraining compared to pretraining. Most samples had total coliform counts exceeding 10^3^ MPN/ml pretraining (71%); this proportion decreased to 62% posttraining. The estimated cumulative probability of being in the high category was marginally significantly higher (*p* = 0.0581) pretraining (76%) compared to posttraining (64%). In conclusion, reductions in the detection of some milk quality and safety parameters were observed. However, a considerable proportion of the milk samples were highly contaminated even after the training. Hence, comprehensive and continued risk mitigation strategies are needed to ensure milk safety for consumers.

## Highlights

• Milk from Ethiopia was highly contaminated with indicator and selected pathogenic bacteria.
• Training of dairy farmers was associated with decreased levels of total coliforms.
• Training was also associated with a lower prevalence of *E. coli* and STEC.
• Sustainable measures are needed to ensure the safety of Ethiopian dairy products.

Foodborne diseases (FBDs) are a global public health concern, affecting around 600 million individuals due to physical, chemical, or biological hazards in food (Havelaar et al., 2015, WHO, 2015). Biological hazards, especially foodborne pathogens that cause diarrhea (e.g., norovirus, *Campylobacter* spp., and *Salmonella* spp.), are the leading causes of FBD. In low- and middle-income countries, the economic burden of these illnesses is estimated to be at least 4 billion USD per year (Grace et al., 2020). African countries, including Ethiopia, are highly affected by FBD. In 2017, illnesses caused by nontyphoidal *Salmonella enterica*, *Campylobacter* spp.*,* and enterotoxigenic *Escherichia coli* resulted in an estimated 723 million United States Dollars in economic costs (van Wagenberg & Havelaar, 2023). However, inadequate surveillance and a scarcity of data limit understanding of the public health and economic impact (Gazu et al., 2023).

Dairy products contribute to the overall burden of FBD (Li et al., 2019) with an estimated 4% of the global FBD burden and 12% of the animal-source FBD burden being attributed to milk and milk products (Grace et al., 2020, Kapoor et al., 2023). In Ethiopia, foodborne pathogens such as *Campylobacter* spp., *Salmonella* spp., and Shiga toxin-producing *Escherichia coli* (STEC) have been found in cow’s milk (Sapp et al., 2022) and there have been several reports of pathogenic contamination of milk (Amenu et al., 2019, Farrell, 2021, Mengistu et al., 2023, Mpatswenumugabo et al., 2023, Yirda et al., 2020). Milk can easily be contaminated when produced, handled, transported, and processed unhygienically. For example, the milk can be directly contaminated by the udder and teats of the cow, the milker, the environment, and the equipment used for milking (FSAUK, 2016, Wanjala et al., 2018, Grace et al., 2020). In Ethiopia, milk is often handled unhygienically and rarely meets national or international microbiological standards (Yirda et al., 2020, Mengistu et al., 2023). Furthermore, milk is not commonly pasteurized and is typically consumed raw (Amenu et al., 2019, Farrell, 2021, Mpatswenumugabo et al., 2023).

The microbial quality and safety of milk can be evaluated through the quantification of contamination indicator microorganisms and the direct detection of pathogens of concern (Deddefo et al., 2023, El-ziney, 2018). Several indicator microorganisms have been used for assessing the microbiological quality of raw milk. These include total bacterial count which serves as a universal indicator, reflecting the overall microbial load, general milking hygiene, or udder health (Fereja et al., 2023, Martin et al., 2023). The coliform count which mainly includes genera such as *Escherichia*, *Klebsiella*, *Enterobacter*, and *Citrobacter* indicates fecal and environmental contamination (Martin et al., 2023). Coliforms can be found in a wide range of environments including the intestine of warm-blooded animals and are commonly grouped into total coliform and thermotolerant coliforms; the latter is a subset of the first and its members can ferment lactose with acid and gas production at 44 °C. Thermotolerant coliforms are indicators of fecal contamination and their presence in milk can signify insufficient sanitary conditions as well as the presence of other pathogenic bacteria (Martin et al., 2016). Psychrotrophic bacteria, like *Pseudomonas* spp. and spore-forming bacteria such as *Bacillus* and *Clostridium,* potentially lead to spoilage or defects in dairy products. These tend to occur at low count, and to our knowledge, there are no guidelines yet for their permissible limits in raw milk (Samaržija et al., 2012). For this research, we focused on coliform and fecal coliform counts to gauge both environmental and fecal contamination occurring during the milk production, collection, and handling phases.

The implementation of intervention methods such as food safety training can reduce microbial contamination of milk and milk products (Amenu et al., 2020). Understanding the full impact of such training before its broad application is crucial. While traditional evaluations of food safety training often rely on knowledge, attitudes, and practices or other behavioral changes as indicators of improvement (Young et al., 2019, Amenu et al., 2020, Young et al., 2020), these subjective assessments, though vital, do not directly measure microbial contamination. Microbial assessments pre- and posttraining could provide a quantitative measure of the effectiveness of the training and important data for risk assessments that measure the public health impact of implementing the training (Michaels et al., 2004). This study aims to examine the association between a food safety training intervention and the microbial quality and safety of raw milk produced by smallholder women dairy farmers in Central Ethiopia.

## Materials and methods

**Figure 1:**
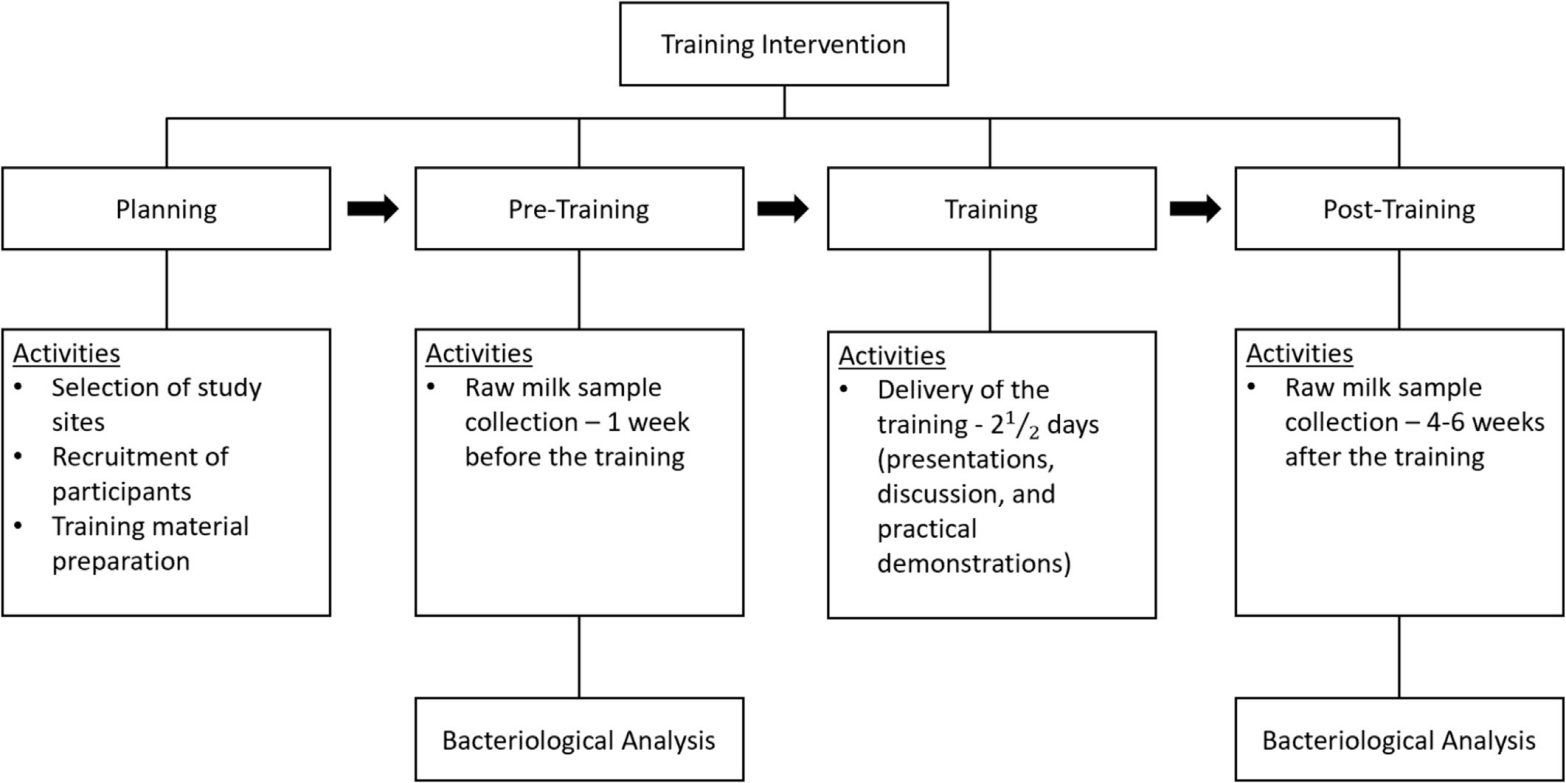
Study design and flow of activities during the training intervention study.

**Study design.** A quasi-experimental prepost study was conducted from January to June 2022 to explore the impact of a 2.5-day training intervention developed and delivered by the “Ensuring Safety and Quality of Milk and Dairy Products Across the Dairy Value Chain of Ethiopia” project on the microbial quality and safety of milk produced by smallholder women dairy farmers in Ethiopia (Fig. 1). The details of the training have been uploaded to Penn State University, College of Agriculture’s website (PSU, 2024). To control the influence of season on bacterial contamination, the study was conducted during the dry season, which is from January through June in Ethiopia.

**Figure 2:**
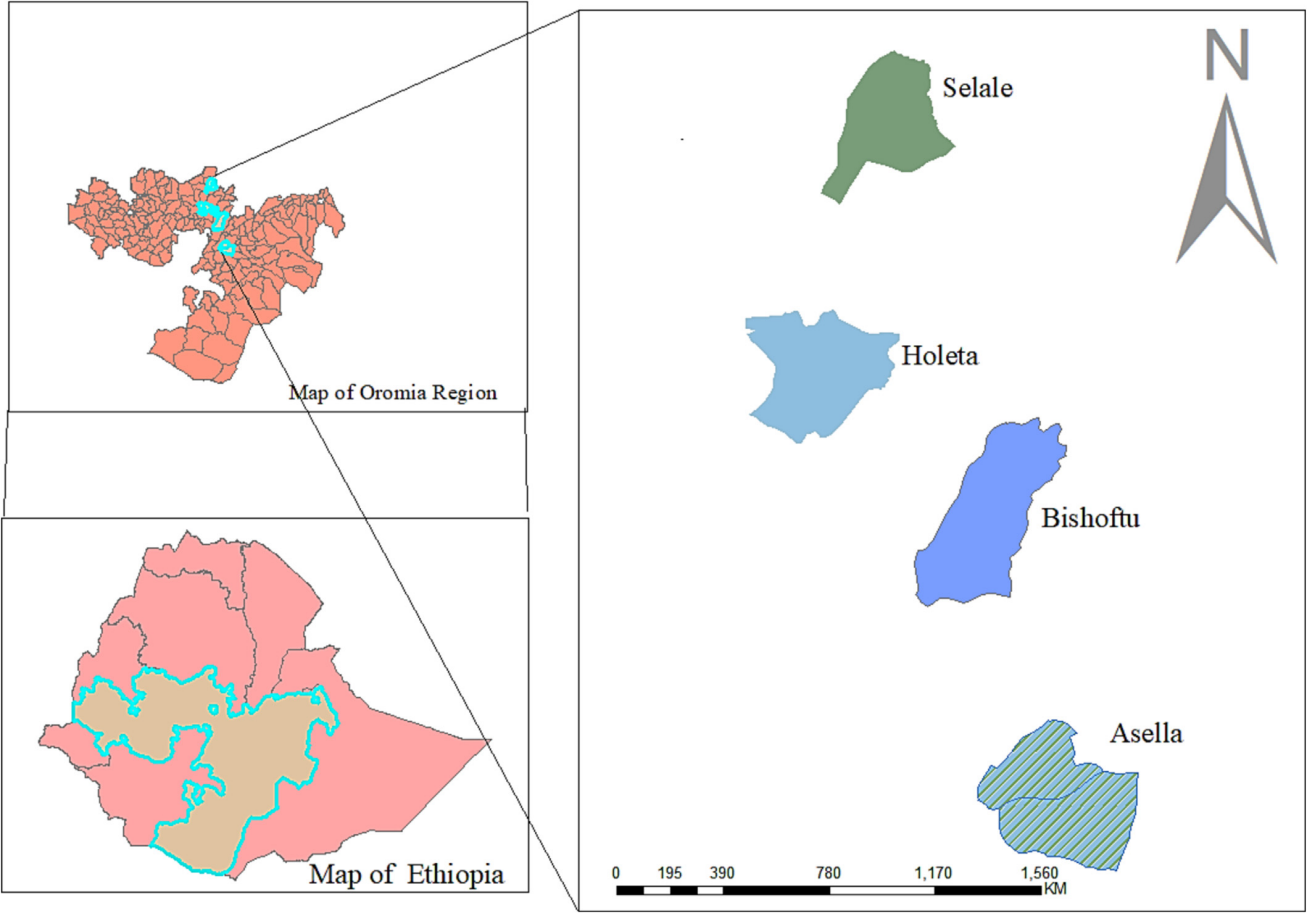
Map of the study area, Central Oromia region, Ethiopia using ArcGIS (https://desktop.arcgis.com/en/arcmap/latest/get-started/installation-guide/installing-on-your-computer.htm).

**Study population and recruitment of participants.** The study population was smallholder women dairy farmers who sell raw milk to milk cooperative unions in four locations (Asella, Bishoftu, Holeta, and Selale) in the central part of Ethiopia (Fig. 2). Farmers who were female; members of a dairy cooperative; worked on a farm owned by their family that holds at least three lactating cows; and largely managed activities on the farm were eligible to participate in the study. Farmers who live outside of Asella, Bishoftu, Holeta, and Selale were excluded from participating in the study. A sample size of 120 women (30 per site) provided 80% power to detect a one-log change (*p* = 0.05) in microbial contamination of the milk, assuming a standard deviation of 2.5 and a drop-out or loss to follow-up proportion of 33%.

Potential participants were randomly selected from an existing list of dairy producers developed by dairy cooperatives and local extension agents in the four study sites. Participants were recruited at milk collection centers in each study site, and written informed consent was obtained in a local language (Amharic or Afaan Oromoo). Ethical approvals were obtained from the University of Gondar (Ref VP/RTT/05/60/2021; Gondar, Ethiopia), the Ohio State University (IRB #2021B042; Columbus, OH, US), and George Washington University (IRB #NCR235227; Washington, DC, US).

**Data collection.** Farms were visited one week before and four to six weeks after the training intervention to collect raw milk samples and interview farmers. At each visit, a volume of 150 ml of raw milk that was ready for sale (typically no more than 15 L) was collected from each farm using a sterile pipette and screw-caped sample collection tube. For the sampling, the raw milk was mixed well, pipetted from the center of the container, and transported to the Ethiopian Public Health Institute, Food Safety Microbiology Laboratory, Addis Ababa, Ethiopia, using a cool box (∼4 °C). The sample was analyzed immediately upon delivery to the laboratory for total coliforms, thermotolerant coliforms, *E. coli*, STEC, *S. enterica,* and *C. jejuni*. Farmers also completed a questionnaire via a face-to-face interview on their knowledge, attitudes, and practices related to milk quality and safety, and an audit/inspection checklist was completed (results not presented here).

**Estimation of coliform counts**. To estimate the total coliforms in the milk sample, the Most Probable Number (MPN) technique was used (Salman and Hamad, 2011, Diane and Mirlei, 2012). Briefly, the milk sample was tenfold serially diluted in triplicates. Aliquots (1 ml) of each dilution were transferred to three dilution tubes, each containing 10 ml sterile MacConkey broth (Oxoid Ltd, UK) and an inverted Durham tube. Inoculated tubes were then incubated at 37 °C for 48 h. The tubes showing acid and gas production were counted and interpreted using a standard 3-tube MPN Table (FDA, 2006) and reported as MPN of total coliforms/ml of milk. If all three dilutions produced negative MPN tubes, the coliform count in the milk sample was considered undetectable.

To estimate thermotolerant coliforms, a loop full of the contents of each of the positive coliform tubes was transferred to a new tube that contained fresh sterile 10 ml MacConkey’s broth and an inverted Durham tube; inoculated tubes were incubated at 44 °C for 48 h. The result was interpreted by counting the tubes that developed acid and gas using the standard 3-tube MPN table (FDA, 2006) and was reported as thermotolerant (fecal) coliform/ml of milk sample. Similarly, if all inoculated tubes were negative, thermotolerant coliforms in the milk sample were considered undetectable.

**Isolation of *Escherichia coli*.***E. coli* was isolated using a standard procedure (Vimont and Delignette-Muller, 2006, ISO7218, 2007). Briefly, about 25 ml of milk samples were mixed with 225 ml of coliform selective broth (Oxoid Ltd, UK) and incubated first at 37 °C for 5 h and then for 36 h at 42 °C. A loop full of enriched samples was then plated on MacConkey agar (Oxoid Ltd, UK). Lactose fermenting and well-isolated three to five colonies on MacConkey agar were streaked onto eosin methylene blue agar (Neogen Culture Media, UK) and then subcultured onto tryptic soy agar (TSA) (Sigma-Aldrich Chemie GmbH, Germany) (Xi et al., 2015). Biochemical tests (indole, motility, citrate, and Hydrogen Sulfide production) were used to screen and identify the presumptive *E. coli* isolates, which were further verified by the Analytical Profile Index (API) 20E testing according to the manufacturer’s instruction (Biomerieux VWR, France).

**Isolation of *Salmonella enterica.****Salmonella* was isolated using a standard procedure (ISO 6579-1, 2017). Briefly, 25 ml of milk samples was mixed with 225 ml of buffered peptone water and incubated at 37 °C for 24 h. Secondary (selective) enrichment was achieved by transferring 0.1 ml from the primary enriched fluid to 10 ml sterile Rappaport-Vassiliadis with soya (RVS) broth (Oxoid, England) and 1.0 ml to 10 ml Muller-Kauffman Tetrathionate novobiocin broth (MKTTn) (Oxoid, England). The secondary enrichment tubes were incubated at 41.5 °C for RVS and 37 °C for MKTTn media. After 24 h of incubation, a loop full of each selective enrichment was plated on xylose lysine deoxycholate agar (Oxoid, England) and hektoen enteric agar (Oxoid, England), which were incubated at 37 °C for 24 h. Well-isolated and presumptive *Salmonella* colonies were subcultured on TSA. The colonies on TSA were biochemically screened using urease, triple sugar iron agar, and lysine decarboxylase tests and further characterized by the API 20E test (Biomerieux VWR, France).

**Isolation of *Campylobacter jejuni.****C. jejuni* was isolated using a modified version of the methods described by El-zamkan & Hameed (2016). Briefly, 25 ml of raw milk samples were centrifuged at 20,000*g* for 40 min and the cell pellets were resuspended in 2.5 ml buffered peptone water. After vortex mixing, 22.5 ml of Bolton broth was added and incubated for 4 h at 37 °C under micro-aerobic conditions (using CampyGen® gas generating kit) for preenrichment. Further enrichment was performed by extending the incubation at 42 °C for 24 h under micro-aerobic conditions. Isolation of colonies was performed by plating on modified charcoal cefoperazone deoxycholate agar (mCCDA) which is *Campylobacter* selective agar that has a selective supplement and incubating for 48 h at 42 °C under micro-aerobic conditions. Characteristic colonies were subjected to biochemical tests. Colonies that produced Gram-negative, spiral, or “S” shaped cells, catalase and oxidase positive reactions, and hydrolysis of Hippurate were considered presumptive *C. jejuni*.

**Confirmation of pathogen by PCR.** The DNA of the three isolated presumptive pathogens (STEC, *S. enterica*, and *C. jejuni*) was extracted by the thermal lysis method (Siddiky et al., 2021, Vondrakova et al., 2014). Briefly, 250 µl of the overnight incubated broth culture was centrifuged at 11,000 rpm for 3 min. The supernatant was discarded, and pellets were washed with 1,000 µl sterile physiological (0.85%) saline. Subsequently, 100 µl of nuclease-free water was added to the pellet, which was then vortexed to homogenize, boiled at 100 °C for 10 min, and chilled on ice for 5 min. Finally, cell debris was separated by centrifugation at 13,500 rpm for 5 min and the supernatant was stored at −20 °C for use as the DNA template.

The presumptive *E. coli* isolates were screened for Shiga toxin and intimin coding genes (*stx1*, *stx2*, and *eae*, respectively) (Table 1). The amplification of these genes was carried out in a total volume of 20 μl reaction containing 10 μl of Master mix (GoTaq colorless Master mix, Promega USA), 0.7 μl of 10 μM each primer (total 4.2 μl), 3.8 μl nuclease-free water, and 2 μl template DNA. The genes were amplified in a thermocycler machine (MasterCycler, China), which was adjusted at initial denaturation of 95 °C for 5 min, with 30 cycles consisting of denaturation at 95 °C for 60 s, annealing 58 °C for 30 s, and extension at 72 °C for 80 s and then followed by a final extension at 72 °C for 5 min. An isolate was considered positive for STEC if it was positive for either *stx_1_* or *stx_2_* genes (Etcheverría & Padola, 2013).

**Table 1:** Target genes, primers, and amplicon length for PCR confirmation of the isolated milk-borne pathogens

Target pathogen for confirmation	Target gene	Primer^a^ pair (5′–3′)	Amplicon length	Reference
*Salmonella* e*nterica*	*invA*	F: 5′-GCTGCGCGCGAACGGCGAAG	389	Ferretti et al. (2001)
		R: 5′-TCCCGGCAGAGTTCCCATT		

Shiga toxin-producing *Escherichia coli*	*stx_1_*	F: 5′-ACACTGGATGATCTCAGTGG	614	Fagan et al. (1999)
		R: 5′-CTGAATCCCCCTCCATTATG		
	*stx_2_*	F: 5′-CCATGACAACGGACAGCAGTT	779	
		R: 5′-CCTGTCAACTGAGCAGCACTTTG		
	*eae*	F: 5′-GTGGCGAATACTGGCGAGACT	890	
		R: 5′-CCCCATTCTTTTTCACCGTCG		

*Campylobacter jejuni*	*hipO*^b^	F: 5′-TGCACCAGTGACTATGAATAACGA	124	Vondrakova et al. (2014)
		R: 5′-TCCAAAATCCTCACTTGCCATT		

a Primer sequences for STEC and *S. enterica* were used in conventional PCR for confirmation.

b Primer sequences of *hipO* gene were used in real-time PCR for *C. jejuni* confirmation; F, forward; R, reserve.

*S. enterica* was confirmed by detecting the *invA* gene. The process was carried out in a total volume of 20 μl containing 10 μl of the Master mix (2x GoTaq colorless Master mix, Promega USA), 0.62 μl of 10 μM forward primer, 0.65 μl of 10 μM reverse primer, 6.73 μl nuclease-free water, and 2 μl DNA template (Ferretti et al., 2001). The gene was amplified in a thermocycler machine (MasterCycler, China) which was adjusted at initial denaturation of 95 °C for 5 min, with 30 cycles consisting of denaturation at 95 °C for 60 s, annealing at 62 °C for 40 s, and extension at 72 °C for 50 s and then followed by the final extension of 5 min at 72 °C.

The amplified products for STEC and *S. enterica* were visualized after gel-electrophoresis using 1% agarose gel, and a 100-bp DNA ladder (Quick-Load ® Purple DNA Ladder; Biolabs*,* England) was used as a molecular size marker to estimate the size of the PCR products. Amplicons were run at 140 V for 50 min and visualized under an ultraviolet transilluminator; then, the image was taken using the gel documentation system (BioTop, gel documentation system, China).

The *C. jejuni* isolates were confirmed using real-time quantitative PCR (qPCR; 7900HT Fast Real-Time PCR System; Applied Biosystems) and *Campylobacter*-specific primers (Table 1). For confirmation of *C. jejuni,* the gene *hipO* was used (Vondrakova et al., 2014). The reaction mixture consisted of 5 µL template DNA, 10 µL SYBR Select Master Mix (Applied Biosystems), 1 µL of each primer (final concentration of 400 nM), and 3 µL nuclease-free water and a total reaction volume of 20 µL. The real-time PCR cycling conditions were 50 cycles of denaturation at 95 °C for 10 s and extension/annealing at 50 °C for 60 s.

**Data analysis.** Data were summarized using descriptive statistics, including frequency tables and empirical histograms. The prevalence of indicator and pathogenic bacteria was estimated by dividing the number of confirmed positives by the total number of samples analyzed, and 95% confidence intervals (CIs) were constructed using the Clopper-Pearson (Exact) method. McNemar’s test for paired data was used to compare the proportion of positive samples pre- and posttraining.

The primary outcomes of interest were total and thermotolerant coliform counts pre- and posttraining. Data followed a bimodal distribution with overdispersion and, as such, were categorized as high (≥1,001 MPN/ml), medium (101–10^3^ MPN/ml), low (21–10^2^ MPN/ml), or very low (≤20 MPN/ml) microbial load for analysis; category ranges were set based on the nature of the data and in a way to avoid numeric gaps between categories. Separate generalized linear mixed models were used to assess differences between total and thermotolerant coliform counts pre- and posttraining (Appendices A and B). This approach allowed us to account for the repeated measures taken from each dairy farm. The models were fit to a multinomial distribution using a cumulative logit link function to account for the categorical nature of the response variables. In both models, the linear predictor included time point (pre- versus posttraining intervention) as a fixed effect and the blocking structure of the farm as a random effect. Location was initially included in the model as a random effect but was removed since the Chi-square divided by the degrees of freedom was greater than one, indicating that the sources of variability were not properly specified. The method of estimation was maximum likelihood implemented through a Laplace approximation. For each response variable, cumulative probability estimates and corresponding 95% CIs were obtained from the fitted models for each ordered category. Wald-based type III tests for fixed effects were used to assess treatment effects. For all statistical tests, a threshold *p* value ≤0.05 was used for significance. A sensitivity analysis using high (≥1,001 MPN/ml), medium (101–10^3^ MPN/ml), and low (0–10^2^ MPN/ml) as categories for the microbial load yielded similar results (not shown).

Data were collected using REDCap electronic data capture tools hosted at The Ohio State University (National Center for Advancing Translational Sciences, Grant UL1TR001070) (Harris et al., 2009, Harris et al., 2019), managed using Excel (Version 2022, Microsoft, Redmond, WA), and analyzed using SAS (version 9.4, SAS Institute, Cary, NC). Data visualization was performed using the ggplot2 package in R (Version 4.4.0, https://www.r-project.org/index.html). Data dictionaries and the raw data will be uploaded and available to the public.

## Results

A total of 120 smallholder women dairy farmers were recruited into the study across the four study sites and participated in the training (Table 2). After the training intervention, five farms failed to complete the follow-up because farmers moved to a different location (*n* = 3) or cows were no longer producing milk (*n* = 2). The mean and median ages of farmers were 42 and 40 years, respectively. Most farmers had completed either primary or secondary education (66%), lived in rural or *peri*-urban areas (63%), and had not received prior training (43%).

**Table 2:** Socio-demographic characteristics of smallholder women dairy farmers (participants) at the initial visit

Characteristics	Categories	Asella (*n* = 30)	Bishoftu (*n* = 30)	Holeta (*n* = 30)	Selale (*n* = 30)	Total (*n* = 120)
**Age**, mean (SD)		42 (12)	43 (17)	44 (9)	37 (10)	41 (12)

**Education,***n* (%)						
	No formal education	2 (7)	6 (20)	7 (23)	4 (13)	19 (16)
	Basic (adult) education	2 (7)	6 (20)	5 (17)	1 (3)	14 (12)
	Primary	10 (33)	10 (33)	8 (27)	11 (37)	39 (32)
	Secondary	14 (46)	7 (23)	9 (30)	11 (37)	41 (34)
	College and above	2 (7)	1 (3)	1 (3)	3 (10)	7 (6)

**Residential area,***n* (%)						
	Urban	11 (37)	15 (50)	7 (23)	11 (37)	44 (37)
	Peri-Urban	17 (56)	0 (0)	3 (10)	3 (10)	23 (19)
	Rural	2 (7)	15 (50)	20 (67)	16 (53)	53 (44)

**Prior dairy training,***n* (%)						
	Yes	0 (0)	20 (67)	14 (47)	6 (20)	40 (33)
	No	2 (7)	10 (33)	16 (53)	24 (80)	52 (43)
	Unknown	28 (93)	0 (0)	0 (0)	0 (0)	28 (24)

If the participant did not provide any information for the sociodemographic characteristic; they were included in the “Unknown” category.

**Figure 3:**
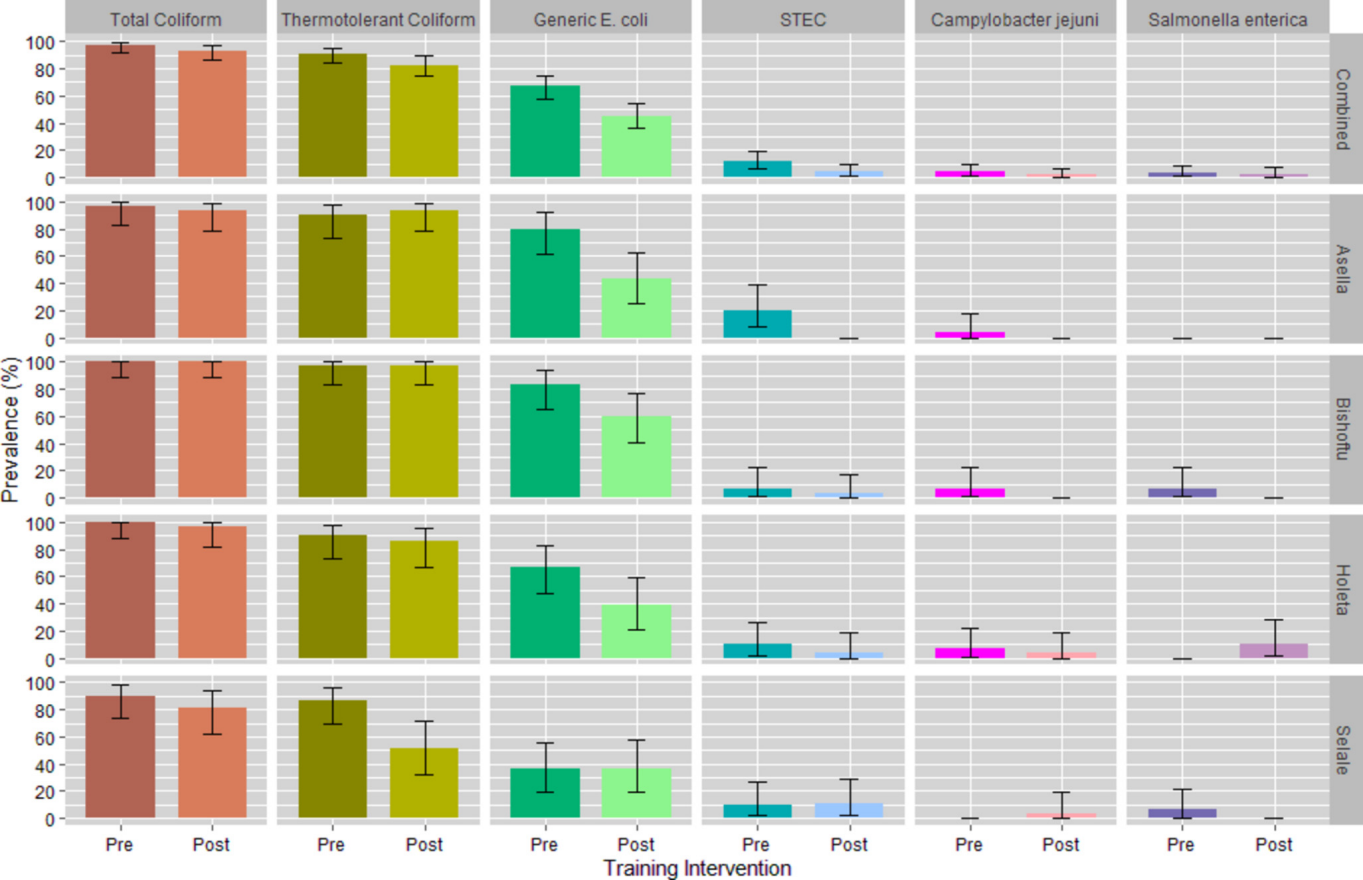
Prevalence and 95% Clopper-Pearson (Exact) Confidence Intervals of total coliforms, thermotolerant coliforms, and *E. coli*, Shiga-toxin producing *E. coli* (STEC), *Campylobacter jejuni*, and *Salmonella enterica* in raw milk samples collected pre- and post-training intervention overall and by study site. Pretraining [Combined (*n* = 120); Asella (*n* = 30); Bishoftu (*n* = 30); Holeta (*n* = 30); Selale (*n* = 30)]. Posttraining [Combined (*n* = 115); Asella (*n* = 30); Bishoftu (*n* = 30); Holeta (*n* = 28); Selale (*n* = 27)].

A total of 120 and 115 raw milk samples were, respectively, collected pre- and posttraining, and analyzed for the presence of total coliforms, thermotolerant coliforms, *E. coli*, STEC, *S. enterica,* and *C. jejuni*. The prevalence of total and thermotolerant coliforms was high across all study sites (Fig. 3, Appendix C) but was not statistically different. The prevalence of generic *E. coli* was 67% (95% CI: 57%, 75%) pretraining and 45% (95% CI: 36%, 55%) posttraining, a difference that was statistically significant (*p* = 0.0389). The prevalence of pathogenic bacteria was substantially lower pre- and posttraining. The prevalence of STEC was 12% (95% CI: 7%, 19%) pretraining and 4% (95% CI: 1%, 10%) posttraining, a difference that was statistically significant (*p* = 0.0005). Among the STEC-positive samples, 63% were positive for *stx_1_*, 37% were positive for *stx_2_* and 11% were positive for *eae*. The prevalence of *S. enterica* was 3% (95% CI: 1%, 8%) pretraining and 2% (95% CI: 1%, 7%) posttraining, a difference that was not statistically significant. The prevalence of *C. jejuni* was 4% (95% CI: 1%, 9%) pretraining and 2% (95% CI: 0%, 6%) posttraining, a difference that was also not statistically significant.

**Figure 4:**
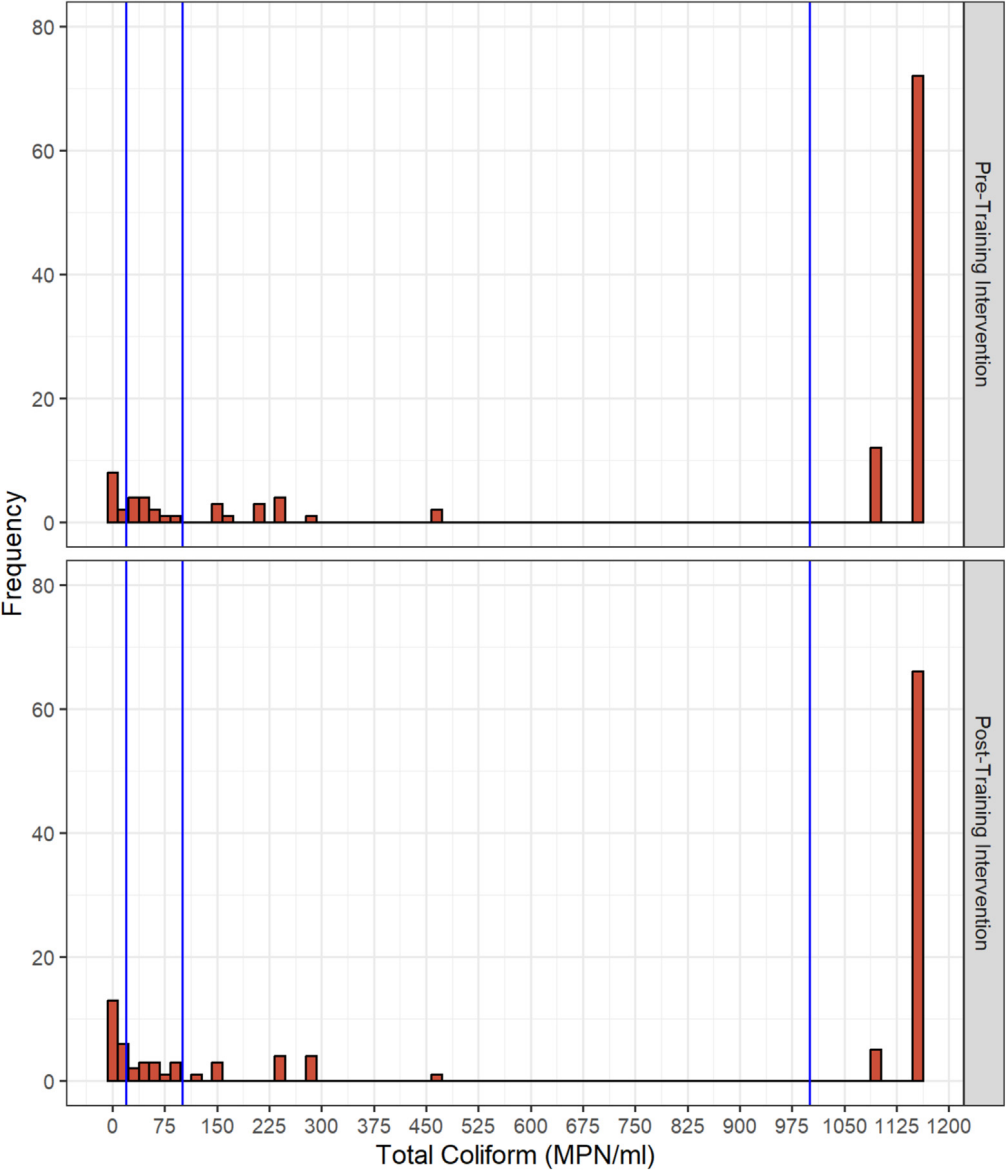
Total coliform counts (MPN/ml) distribution of the raw milk samples collected at the pre- and posttraining intervention. The vertical lines correspond to the boundaries of the data categorically, as very low (≤20 MPN/ml), low (21–10^2^ MPN/ml), medium (101–1,000 MPN/ml), and high (≥1,001 MPN/ml). Based on the most probable number table used (FDA, 2006), undetected and >1,100 MPN/ml were represented as 0 and 1,150 MPN/ml in the histograms.

**Figure 5:**
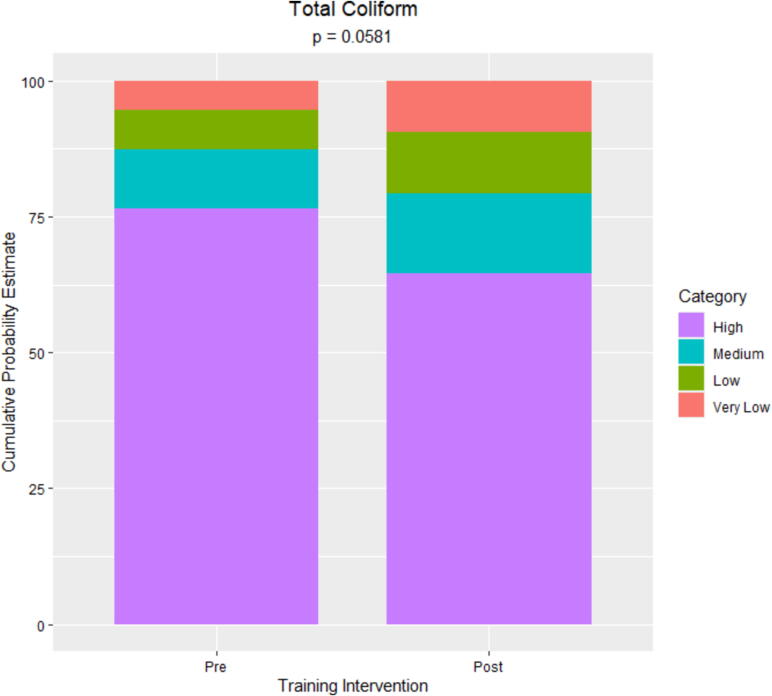
Cumulative probability estimates for total coliform count categories in raw milk samples collected pre- versus posttraining intervention (*n* = 115). Categories were defined as high (≥1,001 MPN/ml), medium (101–10^3^ MPN/ml), low (21–10^2^ MPN/ml), or very low (≤20 MPN/ml) with high serving as the reference category.

Total coliform counts ranged from undetected to more than 1,100 MPN/ml pre- and posttraining (Fig. 4). The majority (71%) of samples had counts exceeding 10^3^ MPN/ml pretraining. Posttraining, this proportion decreased to 62% and the proportion of samples less or equal to 20 MPN/ml increased from 8% to 16%. The estimated cumulative probability of raw milk samples being in the high category was 77% (95% CI: 65%, 85%) pretraining compared to 64% (95% CI: 52%, 75%) posttraining (Fig. 5). This shift demonstrates a reduction in total coliform levels following the training.

**Figure 6:**
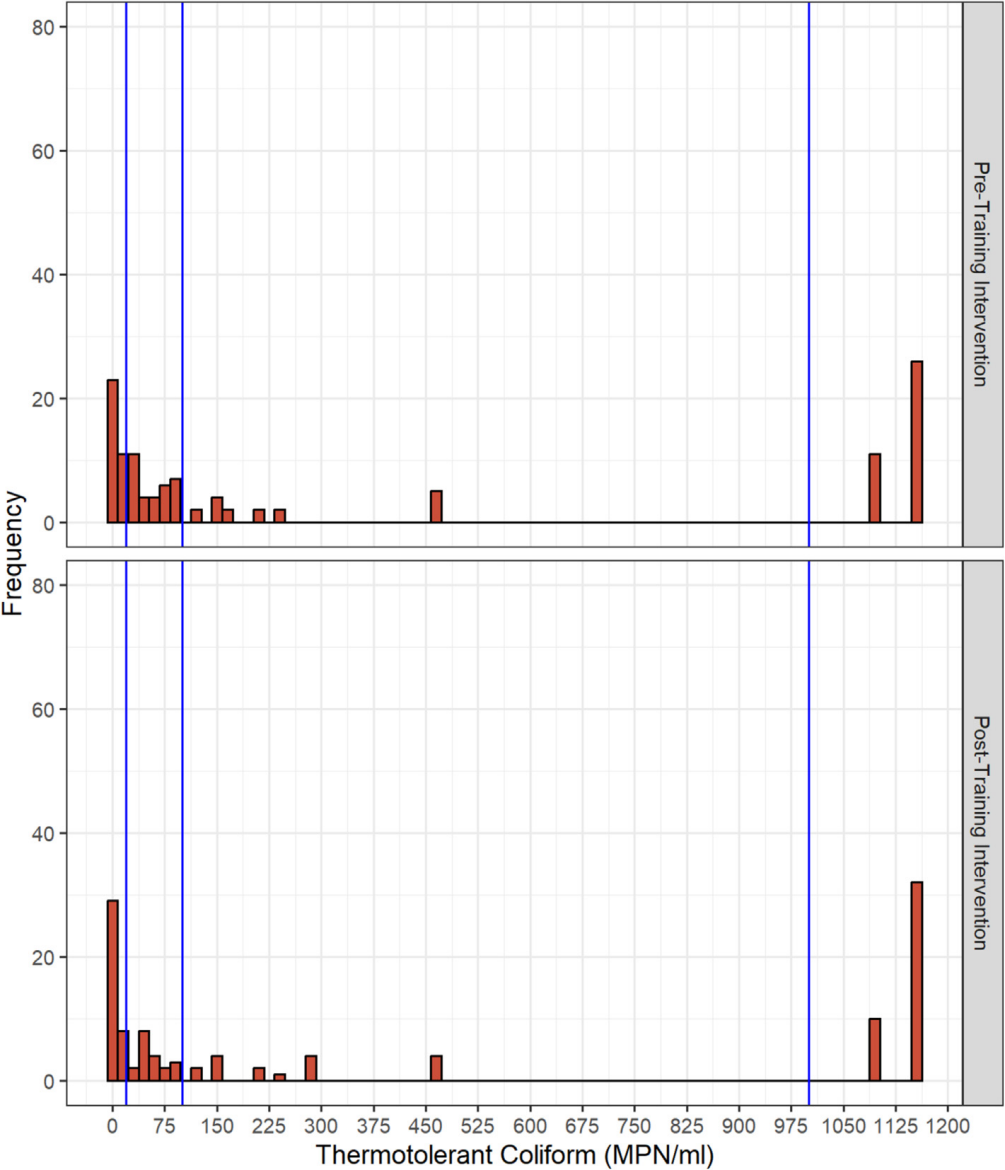
Thermotolerant coliform counts (MPN/ml) distribution of the raw milk samples collected at the pre- and posttraining intervention. The vertical lines correspond to the boundaries of the data categorically, as very low (≤20 MPN/ml), low (21–10^2^ MPN/ml), medium (101–10^3^ MPN/ml), and high (≥1,001 MPN/ml). Based on the most probable number table used (FDA, 2006), undetected and >1,100 MPN/ml were represented as 0 and 1,150 MPN/ml in the histograms.

**Figure 7:**
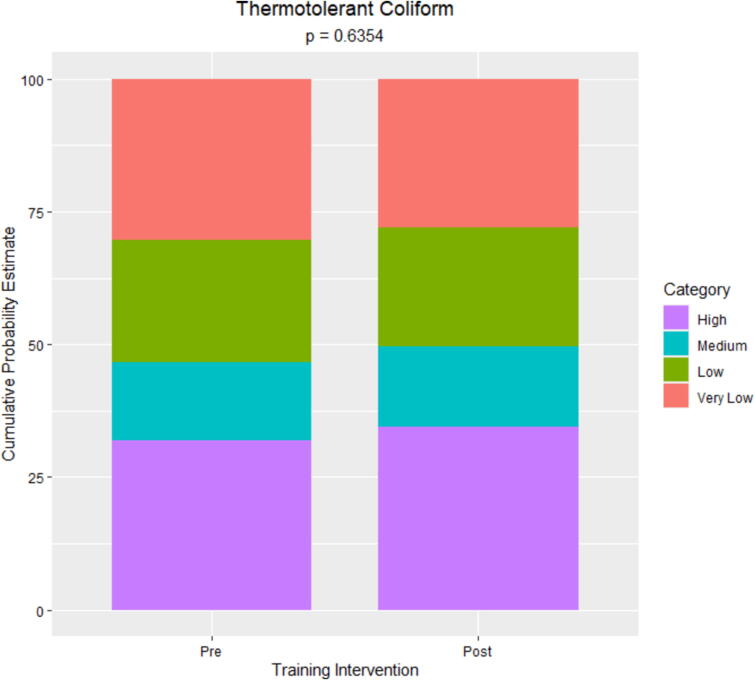
Cumulative probability estimates for thermotolerant coliform count categories in raw milk samples collected pre- versus posttraining intervention (*n* = 115). Categories were defined as high (≥1,001 MPN/ml), medium (101–10^3^ MPN/ml), low (21–10^2^ MPN/ml), or very low (≤20 MPN/ml) with high serving as the reference category.

Thermotolerant coliform counts ranged from undetected to more than 1,100 MPN/ml pre- and posttraining (Fig. 6). Around one-third (32%) of samples had counts exceeding 10^3^ MPN/ml pretraining compared to 36% posttraining. The proportions of samples having counts less than or equal to 20 MPN/ml were similar pretraining (29%) and posttraining (31%). The estimated cumulative probability of raw milk samples being in the high category was 32% (95% CI: 24%, 41%) pretraining compared to 34% (95% CI: 26%, 44%) posttraining, a difference that was not statistically significant (Fig. 7).

## Discussion

To our knowledge, this is the first study to assess the microbiological quality and safety of raw milk produced by women dairy farmers in Central Ethiopia before and after a food safety training. We found reductions in total coliform counts, the prevalence of generic *E. coli*, and STEC pre- to posttraining intervention. However, a considerably high proportion of raw milk samples had maximum counts (>1,100 MPN/ml) of total and thermotolerant coliform pre- and posttraining.

Previous baseline studies have shown that raw milk samples are highly contaminated with total coliform bacteria in Ethiopia and fail to comply with the Ethiopian Standard Authority (ESA) standards for total coliform counts, which is consistent with our results (Mengistu et al., 2023, Nahusenay et al., 2023). According to ESA standards, total coliform counts should not exceed 1000 cfu/ml in raw milk (ESA, 2021). However, the majority of raw milk samples collected from four regions of Ethiopia (Oromia, Southern Nations Nationalities and Peoples (SNNP), Amhara, and Tigray) had total coliform counts that exceeded this level (Mengistu et al., 2023). Similarly, Nahusenay et al. (2023) found high bacterial count in raw milk samples collected from three regions of the country (Oromia, SNNP, and Amhara regions), especially in the wet season. Notably, these studies used direct count (Colony Forming Units (CFU)/ml) rather than the estimation method (MPN/ml) employed in our study to report total coliform concentration. While studies have found a positive correlation between CFU/ml and MPN/ml (Cho et al., 2010), it is challenging to directly compare with our results because a high proportion of the samples were censored (>1,100 MPN/ml) pre- and posttraining. However, regardless of the method to determine the total coliform concentration, samples with a high microbial load of total coliforms suggest a lack of compliance with sanitation standards.

As in other studies, bacteria indicative of poor hygienic practices at the farm level were detected pre- and posttraining. The proportion of samples positive for *E. coli*, a common nonpathogenic bacterium that can also be associated with the presence of pathogenic bacteria, after the training (45%) was similar to those reported in cross-sectional studies conducted in the Ethiopian towns of Bishoftu (42%) (Megersa et al., 2019) and Dessie (55%) (Abebe et al., 2023) but higher than a cross-sectional study conducted in Mekelle (25%) (Yohannes, 2018). Similarly, STEC was detected in 12% of milk samples which aligns with a previous survey conducted in Addis Ababa, Ethiopia (16%5) (Abunna et al., 2019) but higher than that found in a cross-sectional study conducted in Dessie (6%) (Abebe et al., 2023). Prior studies have found that the prevalence of *Salmonella* in the milk at the farm level ranges from zero (Mekonnen et al., 2022, Ndahetuye et al., 2020) to 20% (Bedassa et al., 2023). In this study, the prevalence of *S. enterica* was 3% pre- and 2% posttraining intervention which is comparable to the prevalence in a cross-sectional study conducted in Central Ethiopia (5%) (Geletu et al., 2022) but lower than those reported in studies conducted in Southern Ethiopia (9%) (Asefa et al., 2023) and three other Ethiopian regions (Amhara, Oromia, and SNNP) (20%) (Bedassa et al., 2023). The observed differences across regions could be attributed to differences in dairy farm hygiene practices and the influence of local environmental factors such as temperature and humidity (Toghdory et al., 2022).

There are limited studies on the microbial quality and safety of raw milk pre- and posttraining interventions targeted to smallholder dairy farms globally, and none have been conducted in Ethiopia. A study focusing on the evaluation of a pre- and postfood safety training intervention targeting three milking goat farms in Sao Paulo, Brazil, found no difference in total coliform counts pre- and postintervention, which may not be surprising given a lack of adherence to appropriate handling practices observed on the farms (Tavolaro & Oliveira, 2006). Another study in Negeri Sembilan, Malaysia, found an empirical decrease in total coliform counts in raw milk samples collected before and immediately after training on good agricultural practices (Lee et al., 2017). In our study, we observed a marginally significant difference in total coliform counts pre- and posttraining intervention and observed decreases in the prevalence of STEC, suggesting that the training may have influenced milk handling and hygiene practices.

There are notable differences between our study and the previous studies that limit our ability to compare results. First, there were differences in the microbiological method used (direct count vs. estimation method), which limits our ability to compare the magnitude of the change in total coliform counts pre- and posttraining intervention. Second, different statistical methods were used to analyze the data, resulting in nontrivial consequences for inference. For instance, correlated data are frequently found in microbial testing when sampling is repeatedly conducted on a specific food matrix (Beczkiewicz & Kowalcyk, 2021). As such, the assumptions of independence and homogeneous variances in traditional methods such as analysis of variance (ANOVA) are frequently violated and, as such, their use could lead to biased estimates. In contrast, our use of a GLMM allowed us to account for the correlation in the data and heterogeneous variances, resulting in more reliable estimates and inferences (Bello & Renter, 2018).

Multiple factors could influence associations between the training intervention and the improved microbial status of raw milk observed in our study. In some areas of Ethiopia, the adoption of milk safety practices, including animal health and general hygienic practices, has been positively associated with training (Feyisa et al., 2024). Despite the willingness of smallholder dairy farmers to adhere to milk safety practices, they still face other challenges that can contribute to slow adherence to those practices, for example, shortage of feed and water, the market of their products, and overall farm management (Lemma et al., 2018). Hence, extension specialists should build the capacity of smallholder dairy farmers in Ethiopia by providing more comprehensive training opportunities. The training should involve the core topic of milk safety practices and other topics that may speed their adherence, such as farm management and financial tools for economic growth.

This study has limitations that should be considered when interpreting results. The main limitation is that there was no control group. Instead, all farms were sampled pre- and posttraining intervention. As a result, any intervention effect is confounded with the effect of time, and causal conclusions are not warranted. Finally, this study only focused on smallholder dairy farms in four locations in Ethiopia; therefore, this study is not representative of the milk production of the whole country and results cannot be generalized beyond the farms involved in this study.

## Conclusions

The current study found significant contamination of raw milk with total and thermotolerant coliforms, generic *E. coli*, STEC, *S. enterica*, and *C. jejuni*. While the total coliform, prevalence of *E. coli* and STEC were lower posttraining intervention, a substantial proportion of the milk samples exceeded safety thresholds for consumption. In the context of these findings, we recommend the implementation of comprehensive intervention strategies to enhance microbial safety in dairy production. These strategies should include continuous safety assessments, mentoring programs, and targeted risk mitigation strategies. To foster adherence to best practices and increase milk safety standards, incentive programs should be established for farms that achieve and maintain superior levels of milk quality and safety. Future studies to assess the effectiveness of interventions should include the presence and level of indicator organisms and pathogenic bacteria in food and environmental samples and include a control group to avoid potential confounders. Such a multifaceted approach may address current deficiencies, and promote sustainable improvements in the microbial safety of the dairy sector.

## CRediT authorship contribution statement

**Achenef Melaku Beyene:** Writing – original draft, Methodology, Formal analysis, Data curation, Conceptualization. **Seleshe Nigatu:** Writing – original draft, Methodology, Data curation, Conceptualization. **Juan C. Archila-Godinez:** Writing – original draft, Visualization, Software, Methodology, Formal analysis, Data curation. **Kebede Amenu:** Writing – review & editing, Supervision, Resources, Methodology, Funding acquisition, Conceptualization. **Barbara Kowalcyk:** Writing – review & editing, Visualization, Validation, Supervision, Resources, Project administration, Methodology, Investigation, Funding acquisition, Conceptualization. **Desalegne Degefaw:** Writing – review & editing, Resources, Project administration, Funding acquisition. **Binyam Mogess:** Writing – review & editing, Resources, Project administration, Funding acquisition, Conceptualization. **Baye Gelaw:** Writing – review & editing, Validation, Supervision, Methodology, Conceptualization. **Mucheye Gizachew:** Writing – review & editing, Visualization, Supervision, Conceptualization. **Araya Mengistu:** Writing – review & editing, Validation, Supervision, Conceptualization. **Ahmed G. Abdelhamid:** Writing – review & editing, Validation, Supervision, Methodology, Conceptualization. **James Barkley:** Writing – original draft, Methodology. **Ahmed Yousef:** Writing – review & editing, Validation, Supervision, Resources, Methodology, Funding acquisition, Conceptualization.

## Declaration of competing interest

The authors declare that they have no known competing financial interests or personal relationships that could have appeared to influence the work reported in this paper.

## Appendix A Supplementary material

Supplementary Data 1 Appendices (A, B, C). The raw data is available online under the title “The Dairy Training Intervention of The Assessment and Management of Risk from Non-typhoidal *Salmonella*, Diarrheagenic *Escherichia coli* and *Campylobacter* in Raw Beef and Dairy in Ethiopia” (TARTARE) Project, Doi: .
