# Supplementary material for: The Impact of Training Intervention on Levels of Indicator Bacteria and Prevalence of Selected Pathogens in Raw Milk From Smallholder Women Dairy Farmers in Central Ethiopia
Source: J Food Prot. 2025 Feb 3;88(2):100446. doi: 10.1016/j.jfp.2024.100446 (PMC11789460; doi:10.1016/j.jfp.2024.100446)
Supplement: Supplementary Data 1 — Appendices (A, B, C). The raw data is available online under the title “The Dairy Training Intervention of The Assessment and Management of Risk from Non-typhoidal Salmonella, Diarrheagenic Escherichia coli and Campylobacter in Raw Beef and Dairy in Ethiopia” (TARTARE) Project, Doi: . [file mmc1.docx]

Appendices

**Appendix A.** Estimated prevalence and 95% Exact Confidence Interval of indicator and pathogenic bacteria in the raw milk samples collected before and after the training intervention by study site

|  |  | **Prevalence % (95% Exact Confidence Interval)** | | | | |
| --- | --- | --- | --- | --- | --- | --- |
|  |  | **Combined**  Pre (*n* = 120)  Post (*n* = 115) | **Asella**  Pre (*n* = 30)  Post (*n* = 28) | **Bishoftu**  Pre (*n* = 30)  Post (*n* = 30) | **Holeta**  Pre (*n* = 30)  Post (*n* = 27) | **Selale**  Pre (*n* = 30)  Post (*n* = 22) |
| Total Coliform | **Pre-training** | 96.67 (91.69; 99.08) | 96.67 (82.78; 99.92) | 100.00 (88.43; 100.00) | 100.00 (88.43; 100.00) | 90.00 (73.47; 97.89) |
|  | **Post-training** | 93.04 (86.75; 96.95) | 93.33 (77.93; 99.18) | 100.00 (88.43; 100.00) | 96.43 (81.65; 99.91) | 81.48 (61.92; 93.70) |
| Thermotolerant Coliform | **Pre-training** | 90.83 (84.19; 95.33) | 90.00 (73.47; 97.89) | 96.67 (82.78; 99.92) | 90.00 (73.47; 97.89) | 86.67 (69.28; 96.24) |
|  | **Post-training** | 82.61 (74.43; 89.04) | 93.33 (77.93; 99.18) | 96.67 (82.78; 99.92) | 85.71 (67.33; 95.97) | 51.85 (31.95; 71.33) |
| Generic *E.coli* | **Pre-training** | 66.67 (57.48; 75.01) | 80.00 (61.43; 92.29) | 83.33 (65.28; 94.36) | 66.67 (47.19; 82.71) | 36.67 (19.93; 56.14) |
|  | **Post-training** | 45.22 (35.92; 54.77) | 43.33 (25.46; 62.57) | 60.00 (40.60; 77.34) | 39.29 (21.50; 59.42) | 37.04 (19.40; 57.63) |
| STEC | **Pre-training** | 11.67 (6.53; 18.80) | 20.00 (7.71; 38.57) | 6.67 (0.82; 22.07) | 10.00 (2.11; 26.53) | 10.00 (2.11; 26.53) |
|  | **Post-training** | 4.35 (1.43; 9.85) | 0.00 | 3.33 (0.08; 17.22) | 3.57 (0.09; 18.35) | 11.11 (2.35; 29.16) |
| *Salmonella* *enterica* | **Pre-training** | 3.33 (0.92; 8.31) | 0.00 | 6.67 (0.82; 22.07) | 0.00 | 6.67 (0.82; 22.07) |
|  | **Post-training** | 2.61 (0.54; 7.43) | 0.00 | 0.00 | 10.71 (2.27; 28.23) | 0.00 |
| *Campylobacter jejuni* | **Pre-training** | 4.17 (1.37; 9.46) | 3.33 (0.08; 17.22) | 6.67 (0.82; 22.07) | 6.67 (0.82; 22.07) | 0.00 |
|  | **Post-training** | 1.74 (0.21; 6.14) | 0.00 | 0.00 | 3.57 (0.09; 18.35) | 3.70 (0.09; 18.97) |

**Appendix B.** Models for total and thermotolerant coliforms

**Eq. (B.1)** Linear predictor

$$\boldsymbol{E(n}_{\boldsymbol{cij}}\boldsymbol{)=}\boldsymbol{g}^{\boldsymbol{-1}}\mathbf{(}\boldsymbol{n}_{\boldsymbol{c}}\boldsymbol{+}\boldsymbol{farm}_{\boldsymbol{i}}\boldsymbol{+}\boldsymbol{\alpha}_{\boldsymbol{j}}\boldsymbol{)}$$

**Eq. (B.2)** Distributions

$$\boldsymbol{y}_{\boldsymbol{cij}}\boldsymbol{|}\boldsymbol{farm}_{\boldsymbol{i}}\boldsymbol{\sim Multinomial(}\boldsymbol{N}_{\boldsymbol{ij}}\boldsymbol{,}\boldsymbol{\pi}_{\boldsymbol{c}}\boldsymbol{)}$$

$$\boldsymbol{farm}_{\boldsymbol{i}}\boldsymbol{\sim i.i.d.N(0,}{\boldsymbol{\sigma}^{\boldsymbol{2}}}_{\boldsymbol{farm}}\boldsymbol{)}$$

**Eq. (B.3)** Links

$$\boldsymbol{h(n}_{\boldsymbol{(high)ij}}\boldsymbol{)=}\frac{\boldsymbol{1}}{\boldsymbol{1+}\boldsymbol{e}^{\boldsymbol{-n}_{\boldsymbol{(high)ij}}}}\boldsymbol{=}\boldsymbol{\pi}_{\boldsymbol{(high)ij}}$$

$$\boldsymbol{h(n}_{\boldsymbol{(medium)ij}}\boldsymbol{)=}\frac{\boldsymbol{1}}{\boldsymbol{1+}\boldsymbol{e}^{\boldsymbol{-n}_{\boldsymbol{(medium)ij}}}}\boldsymbol{=}\boldsymbol{\pi}_{\boldsymbol{(high)ij}}\boldsymbol{+}\boldsymbol{\pi}_{\boldsymbol{(medium)ij}}$$

$$\boldsymbol{h(n}_{\boldsymbol{(low)ij}}\boldsymbol{)=}\frac{\boldsymbol{1}}{\boldsymbol{1+}\boldsymbol{e}^{\boldsymbol{-n}_{\boldsymbol{(low)ij}}}}\boldsymbol{=}\boldsymbol{\pi}_{\boldsymbol{(high)ij}}\boldsymbol{+}\boldsymbol{\pi}_{\boldsymbol{(medium)ij}}\boldsymbol{+}\boldsymbol{\pi}_{\boldsymbol{(low)ij}}$$

Where c corresponds to the count category (*c*=high, medium, low, and very low); *i* corresponds to the individual farm (*i*=1,…,120), and *j* corresponds to the pre- or post-training intervention (*j*=1, 2). The response variable, $\boldsymbol{n}_{\boldsymbol{cij}}$, is the effect of the training intervention on the total or fecal coliform counts categories for each farm. The intercept for the *c^th^* link, $\boldsymbol{n}_{\boldsymbol{c}}$, is treated as a random effect. The effect of the *i^th^* farm, $\boldsymbol{farm}_{\boldsymbol{i}}$, on the response variable is treated as a random effect and blocking structure that is identically, independently and normally distributed with a mean of zero ($\boldsymbol{i.i.d.N(0,}{\boldsymbol{\sigma}^{\boldsymbol{2}}}_{\boldsymbol{farm}}\boldsymbol{)}$). The effect of the *j^th^* time point, $\boldsymbol{\alpha}_{\boldsymbol{j}}$, on the response variable is treated as a fixed effect. From the $\boldsymbol{N}_{\boldsymbol{ij}}$ observations taken, the observations in each count category, $\boldsymbol{y}_{\boldsymbol{cij}}$, have a multinomial distribution with a response probability of $\boldsymbol{\pi}_{\boldsymbol{c}}$ for the *c^th^* category. The inverse link of the cumulative logit, $\boldsymbol{g}^{\boldsymbol{-1}}$, denoted by $\boldsymbol{h(n}_{\boldsymbol{(high)ij}}\boldsymbol{)}$**,** $\boldsymbol{h(n}_{\boldsymbol{(medium)ij}}\boldsymbol{)}$**,** $\boldsymbol{h(n}_{\boldsymbol{(low)ij}}\boldsymbol{)}$, is expressed as the cumulative probability for being in the high, medium, and low category, respectively. Since there are four categories, the three links represent the boundaries between the categories.

**Appendix C.** Generalized Linear Mixed Models (GLMM) code implemented on Statistical Analysis Software (SAS; Version 9.4, SAS Institute, Cary, NC) for total and thermotolerant coliforms

/* GLMM TOTAL COLIFORMS */

**PROC** **GLIMMIX** DATA = check.tartare_dairy METHOD = laplace;

CLASS time_point farm;

MODEL total_coliform_cat = time_point / DIST = multinomial LINK = cumlogit oddsratio;

RANDOM intercept / subject = farm;

ESTIMATE 'total_coliform_cat = tc_0_high, time_point = 1' intercept **1** **0** **0** time_point **1** **0** / ilink cl alpha = **0.05**;

ESTIMATE 'total_coliform_cat = tc_1_medium, time_point = 1' intercept **0** **1** **0** time_point **1** **0** / ilink cl alpha = **0.05**;

ESTIMATE 'total_coliform_cat = tc_2_low, time_point = 1' intercept **0** **0** **1** time_point **1** **0** / ilink cl alpha = **0.05**;

ESTIMATE 'total_coliform_cat = tc_0_high, time_point = 2' intercept **1** **0** **0** time_point **0** **1** / ilink cl alpha = **0.05**;

ESTIMATE 'total_coliform_cat = tc_1_medium, time_point = 2' intercept **0** **1** **0** time_point **0** **1** / ilink cl alpha = **0.05**;

ESTIMATE 'total_coliform_cat = tc_2_low, time_point = 2' intercept **0** **0** **1** time_point **0** **1** / ilink cl alpha = **0.05**;

CONTRAST '2 vs 1' time_point **1** -**1**;

RUN;

/* GLMM FECAL COLIFORMS */

**PROC** **GLIMMIX** DATA = check.tartare_dairy METHOD = laplace;

CLASS time_point farm;

MODEL fecal_coliform_cat = time_point / DIST = multinomial LINK = cumlogit oddsratio;

RANDOM intercept / subject = farm;

ESTIMATE 'fecal_coliform_cat = fc_0_high, time_point = 1' intercept **1** **0** **0** time_point **1** **0** / ilink cl alpha = **0.05**;

ESTIMATE 'fecal_coliform_cat = fc_1_medium, time_point = 1' intercept **0** **1** **0** time_point **1** **0** / ilink cl alpha = **0.05**;

ESTIMATE 'fecal_coliform_cat = fc_2_low, time_point = 1' intercept **0** **0** **1** time_point **1** **0** / ilink cl alpha = **0.05**;

ESTIMATE 'fecal_coliform_cat = fc_0_high, time_point = 2' intercept **1** **0** **0** time_point **0** **1** / ilink cl alpha = **0.05**;

ESTIMATE 'fecal_coliform_cat = fc_1_medium, time_point = 2' intercept **0** **1** **0** time_point **0** **1** / ilink cl alpha = **0.05**;

ESTIMATE 'fecal_coliform_cat = fc_2_low, time_point = 2' intercept **0** **0** **1** time_point **0** **1** / ilink cl alpha = **0.05**;

CONTRAST '2 vs 1' time_point **1** -**1**;

RUN;
